# Chlorhexidine-dress related contact dermatitis—the precautionary principle is no more relevant!

**DOI:** 10.1186/s13054-020-03411-6

**Published:** 2020-12-09

**Authors:** Philippe Eggimann, Jean-Luc Pagani, Jean-Pierre Revelly, Yok-Ai Que

**Affiliations:** 1 Department of Orthopedics and Traumatology, Centre Hospitalier Universitaire Vaudois (CHUV), Rue du Bugnon 46, 1211 Lausanne, Switzerland; 2 Service of Intensive Care Medicine, Centre Hospitalier Universitaire Vaudois, Lausanne, Switzerland; 3 Department of Intensive Care Medicine, Inselspital, Bern University Hospital, University of Bern, Bern, Switzerland

Dear editor,

We read carefully the Buetti et al. [1] post hoc analysis of two open randomized multicenter French studies, comparing non-disinfectant dressings with CHG-releasing sponge (December 2006 to May 2008) [2] and with CHG-releasing gel (May 2010 to July 2011) [3].

After adjustment for confounders, gel-dress showed similar risk for MCRI compared to sponge-dress (HR 0.80, 95% CI 0.28–2.31, *p* = 0.68) and CRBSI (HR 1.13, 95% CI 0.34–3.70, *p* = 0.85), less dressing disruptions (OR 0.72, 95% CI 0.60–0.86, *p* < 0.001), and more contact dermatitis (OR 3.60, 95% CI 2.51–5.15, *p* < 0.01). However, gel-dress increased the risk of contact dermatitis only if CHG was used for skin antisepsis (OR 1.94, 95% CI 1.38–2.71, *p* < 0.01).

Alcoholic-CHG was used for skin preparation in 1533 of gel-dress recipients (72.7%) and only 20 patients with sponge-dress (1.3%) [1]. In a recently published real-world evidence study, the gel-dress was applied with CHG for skin antisepsis [4]. Initial rates of contact dermatitis were consistent with previous findings [3]. Following the implementation of a redesigned dressing in March 2012, contact dermatitis rates dropped from 5.5 episodes/1000 device-days to 0.3/1000 device-days and same levels were reported for both sponge- and the gel-dress [4].

Several factors may have played a role in this improvement. Firstly, re-designed adhesive distribution in the dressing resulted in an improved skin moisture evaporation through the transparent membrane. Secondly, two-step skin preparation (scrubbing) were never incorporated in our guidelines for skin antisepsis with CHG. Thirdly, time allowed for the disinfecting solution to fully evaporate was part of our guidelines at time of introduction of CHG dressings in 2007. This practice may decrease initial moisture build-up on the skin surface.

Above-mentioned conclusion on increased risk for skin irritation is highly concerning as it may challenge major elements from widely recommended bundle to prevent CRBSI. Alcoholic solutions of CHG have demonstrated superior efficacy in prevention of infections complications and graded IA by CDC Guidelines [5]. Supported by two randomized studies [1, 2] and confirmed by a real-world study [4], the use of CHG-dressings to reduce CRBSI is also a IA scored recommendation [5].

Accordingly, we congratulate Buetti et al. for their outstanding methodological skills applied in their post hoc analysis, but it is of crucial importance to challenge some of the conclusions. We urge the medical community not to discard either the use of chlorhexidine-dressings and/or alcoholic solutions of CHG for skin preparation.

## Data Availability

Not applicable. No new or original data. All studies mentioned are published and include ethical approval and consent to participate.
